# Robot-assisted versus conventional laparoscopic surgery for endometrial cancer: long-term comparison of outcomes

**DOI:** 10.3389/fonc.2023.1219371

**Published:** 2023-09-15

**Authors:** Kyung Jin Eoh, Tae-Joong Kim, Jeong-Yeol Park, Hee Seung Kim, Jiheum Paek, Young Tae Kim

**Affiliations:** ^1^ Department of Obstetrics and Gynecology, Yongin Severance Hospital, Yonsei University College of Medicine, Yongin, Republic of Korea; ^2^ Department of Obstetrics and Gynecology, Samsung Medical Center, Seoul, Republic of Korea; ^3^ Department of Obstetrics and Gynecology, University of Ulsan College of Medicine, Asan Medical Center, Seoul, Republic of Korea; ^4^ Department of Obstetrics and Gynecology, Seoul National University College of Medicine, Seoul, Republic of Korea; ^5^ Departments of Obstetrics and Gynecology, Ajou University School of Medicine, Suwon, Republic of Korea; ^6^ Department of Obstetrics and Gynecology, Institute of Women’s Medical Life Science, Yonsei Cancer Center, Severance Hospital, Yonsei University College of Medicine, Seoul, Republic of Korea

**Keywords:** endometrial neoplasms, robotic surgical procedures, laparoscopy, mortality, postoperative complications

## Abstract

**Objective:**

There is a lack of multi-institutional large-volume and long-term follow-up data on comparisons between robot-assisted surgery and conventional laparoscopic surgery. This study compared the surgical and long-term survival outcomes between patients who underwent robot-assisted or conventional laparoscopic surgery for endometrial cancer.

**Methods:**

We retrospectively reviewed the data of patients from five large academic institutions who underwent either robot-assisted or conventional laparoscopic surgery for the treatment of endometrial cancer between 2012 and 2017, ensuring at least 5 years of potential follow-up. Intra- and postoperative outcomes, long-term disease-free survival, and overall survival were compared.

**Results:**

The study cohort included 1,003 unselected patients: 551 and 452 patients received conventional laparoscopic and robot-assisted surgery, respectively. The median follow-up duration was 57 months. Postoperative complications were significantly less likely to occur in the robot-assisted surgery group compared to the laparoscopic surgery group (7.74% *vs*. 13.79%, P = 0.002), primarily limited to minor complications. There were no significant differences in survival: 5-year disease-free survival was 91.2% versus 90.0% (P = 0.628) and overall survival was 97.9% versus 96.8% (P = 0.285) in the robot-assisted and laparoscopic surgery cohorts, respectively. Cox proportional hazard regression models demonstrated that the mode of surgery was not associated with disease-free survival (hazard ratio, 0.897; confidence interval, 0.563–1.429) or overall survival (hazard ratio, 0.791; confidence interval, 0.330–1.895) after adjusting for confounding factors.

**Conclusion:**

Robot-assisted surgery for endometrial cancer demonstrates comparable long-term survival outcomes and a reduced incidence of postoperative minor complications when compared to conventional laparoscopic surgery.

## 1 Introduction

Endometrial cancer is the most common malignancy of the female reproductive tract in developed countries (1). Surgery is an essential step in the management of the disease. The need for postoperative adjuvant treatment to minimize recurrence can be determined from the pathological analysis of the surgical specimen (2).

Minimally invasive surgery (MIS) for early stage endometrial cancer offers equivalent survival outcomes with reduced intra- and postoperative morbidity, compared to laparotomic surgery (3, 4). Specifically, the introduction of robot-assisted laparoscopic surgery (RS) has encouraged more gynecologic oncologists to adopt MIS when treating endometrial cancer (5). RS provides potential benefits over conventional laparoscopic surgery (LS), including binocular view, additional degrees of rotational freedom, decreased reliance on skilled assistance, and relatively shallower learning curve. Moreover, RS has been reported as a feasible, safe, and reproducible alternative to LS, even in the cases of obese or elderly patients with endometrial cancer (6, 7). As a result, the proportion of endometrial cancers treated through MIS has gradually increased, approaching 90% at high-volume hospitals, which reflects the increasing use of RS (8, 9). However, there is a lack of large-volume, long-term follow-up data on the direct comparison of oncologic and surgical outcomes between RS and LS for the treatment of endometrial cancer. Currently, most reports suggest that short-term complication rates and survival outcomes for patients with endometrial cancer are similar between RS and LS (10, 11).

This study compared the long-term survival outcomes and surgical outcomes, including intra- and postoperative complications, between patients who underwent RS or LS for endometrial cancer in five high-volume hospitals in Korea.

## 2 Materials and methods

### 2.1 Study population and data collection

This retrospective multicenter study was approved by the institutional review board of Yonsei university of Korea (Approval No. 4-2021-0988). The requirement of informed consent was waived due to the retrospective nature of the study. The study was conducted in accordance with the Declaration of Helsinki. We reviewed the medical records of each institution and identified patients who met the following inclusion criteria: (1) age ≥18 years, (2) pathologically confirmed endometrial cancer, (3) diagnosis made between January 2012 and June 2017, and (4) having undergone laparoscopic or robot-assisted MIS, including total hysterectomy and peritoneal lavage, with or without lymph node biopsy for the treatment of endometrial cancer. Patients were excluded if they met any of the following criteria: (1) immunocompromised or pregnant, (2) synchronous double primary cancers, (3) treated medically or with radiation alone, and (4) lost to follow-up without evidence of disease recurrence or death.

### 2.2 Data analysis

Patients who underwent robot-assisted surgery for endometrial cancer were assigned to the RS group, whereas those who underwent conventional laparoscopic surgery were assigned to the LS group. Differences in the demographic characteristics, comorbidities, and detailed intra- and postoperative outcomes, including complications, details of surgical procedures, results of pathological analysis, and survival outcomes were compared between the RS and LS groups. Disease-free survival (DFS) was defined as the duration from the date of surgery to the recurrence or end of follow-up. Overall survival (OS) was defined as the interval between the date of surgery and death or the end of follow-up, whichever came first.

Patients were classified into subgroups based on the number of ports utilized during the procedure. An analysis of subgroups was conducted to evaluate and compare the surgical outcomes and survival rates between patients who underwent multiport laparoscopy (mLS), single port laparoscopy (sLS), multiport robot-assisted surgery (mRS), and single port robot-assisted surgery (sRS).

### 2.3 Statistical analysis

Demographic, surgical, and pathological characteristics were compared using paired t-tests and chi-square tests. Kaplan–Meier methods with log-rank tests were used for survival analysis. Multivariable Cox proportional hazards models were used to determine the hazard ratio (HR) to predict recurrence or death after adjusting for confounding variables. All statistical analyses were conducted using the IBM SPSS Statistics software (version 26.0; SPSS Inc., Chicago, IL, USA).

## 3 Results

A total of 1,003 patients who underwent surgical treatment for endometrial cancer during the study window were identified from five participating centers: 551 (54.9%) and 452 (45.1%) patients underwent LS and RS, respectively. Based on the number of ports, 456 (45.5%), 95 (9.5%), 419 (41.8%), and 33 (3.3%) patients were classified into the mLS, sLS, mRS, and sRS groups, respectively. The average age of the patients was 55 years at the time of surgery, and the RS group was significantly younger, with fewer overall comorbidities and a more favorable performance status as compared with the LS group. There were no significant differences in body mass index. Patient characteristics, including comorbidities and performance status, are presented in **Table 1**. Patient characteristics classified according to the number of ports utilized are demonstrated in **Supplementary Table 1**.

**Table 1:** Patient characteristics.

	Total (n = 1003)	%, SD	LS (n = 551)	%, SD	RS (n = 452)	%, SD	*P*-value
Age, years, median (range)	55 (23–86)		55 (23–86)		53 (33–72)		<0.001
BMI, kg/m^2^, mean (SD)	24.349	3.94	24.303	3.99	24.474	3.86	0.076
Comorbidity	299	29.81	183	33.21	116	25.66	0.009
Angina	2	0.20	2	0.36	0	0.00	
MI	2	0.20	2	0.36	0	0.00	
Afib	1	0.10	1	0.18	0	0.00	
Stroke	5	0.50	4	0.73	1	0.22	
CVD	10	1.00	5	0.91	5	1.11	
PVD	2	0.20	1	0.18	1	0.22	
COPD	3	0.30	1	0.18	2	0.44	
CPD	10	1.00	5	0.91	5	1.11	
LC	8	0.80	5	0.91	3	0.66	
Dyslipidemia	63	6.28	37	6.72	26	5.75	
HTN	220	21.93	133	24.14	87	19.25	
DM	63	6.28	44	7.99	19	4.20	
Rheumatologic disease	4	0.40	4	0.73	0	0.00	
Dementia	2	0.20	1	0.18	1	0.22	
Performance state							
ASA I	417	41.58	204	37.02	213	47.12	<0.001
ASA II	507	50.55	290	52.63	217	48.01	
ASA III	75	7.48	53	9.62	22	4.87	
ASA IV	4	0.40	4	0.73	0	0.00	

SD, standard deviation; RS, robot-assisted surgery group; LS, conventional laparoscopic surgery group; BMI, body mass index; MI, myocardial infarction; Afib, atrial fibrillation; CVD, cerebrovascular disease; PVD, peripheral vascular disease; COPD, chronic obstructive pulmonary disease; CPD, chronic pulmonary disease; LC, liver cirrhosis; HTN, hypertension; DM, diabetes with chronic complications; ASA, American Society of Anesthesiologists.

Surgical staging, including total hysterectomy and peritoneal lavage with or without lymph node biopsy, was performed for all the included patients. There were no significant differences in the FIGO stage, histologic type, tumor grade, lympho-vascular space invasion (LVSI), invasion depth, and tumor size between the two cohorts (**Table 2**). No significant variations in the pathologic outcomes were observed in relation to the number of ports utilized, as per the data presented in **Supplementary Table 2**.

**Table 2:** Pathologic outcomes.

	Total (n = 1003)	%, SD	LS (n = 551)	%, SD	RS (n = 452)	%, SD	*P*-value
FIGO stage							
IA	754	75.17	406	73.68	348	76.99	0.844
IB	105	10.47	62	11.25	43	9.51	
II	47	4.69	28	5.08	19	4.20	
IIIA	24	2.39	15	2.72	9	1.99	
IIIB	4	0.40	3	0.54	1	0.22	
IIIC1	33	3.29	17	3.09	16	3.54	
IIIC2	29	2.89	17	3.09	12	2.65	
IVB	7	0.70	3	0.54	4	0.88	
Histology							
Endometrioid	899	89.63	486	88.20	413	91.37	0.4
Non-endometrioid	104	10.37	65	11.80	39	8.63	
Tumor grade							
1	609	60.72	333	60.44	276	61.06	0.671
2	249	24.83	139	25.23	110	24.34	
3	137	13.66	73	13.25	64	14.16	
NA	8	0.80	6	1.09	2	0.44	
LVSI	156	15.55	91	16.52	65	14.38	0.722
Invasion depth, cm (SD)	0.451	0.67	0.479	0.64	0.417	0.70	0.486
Tumor size, cm (SD)	2.055	1.88	2.094	1.86	2.006	1.91	0.093

SD, standard deviation; RS, robot-assisted surgery group; LS, conventional laparoscopic surgery group; FIGO, Fédération Internationale de Gynécologie et d’Obstétrique; NA, not available; LVSI, lymphovascular space invasion.

Compared with the LS group, the RS group underwent more complicated procedures, including para-aortic lymph node dissection (54.0% *vs*. 51.2%). Sentinel lymph node sampling was performed more frequently in the RS group as compared with the LS group (58.0% *vs*. 18.7%; *P* < 0.001). Total laparoscopic hysterectomy was performed more frequently in the RS group (76.6% *vs*. 57.9; *P* < 0.001). Most vault sutures were performed using vicryl; however, V-lock sutures were more common in the RS group than in the LS group (17.7% *vs*. 6.7%) (**Table 3**). The operative description in correlation to the number of ports utilized during the procedure is presented in the **Supplementary Table 3**.

**Table 3:** Surgical procedures.

	Total (n = 1003)	%	LS (n = 551)	%	RS (n = 452)	%	*P*-value
Operation type							
Hysterectomy only	5	0.50	3	0.54	2	0.44	<0.001
H+BS	26	2.59	21	3.81	5	1.11	
H+BSO	43	4.29	37	6.72	6	1.33	
H+BSO+BPLD	403	40.18	208	37.75	195	43.14	
H+BSO+BPLD+PALD	490	48.85	255	46.28	235	51.99	
H+BSO+BPLD+PALD+Omentectomy	36	3.59	27	4.90	9	1.99	
Sentinel LN biopsy	365	36.39	103	18.69	262	57.96	<0.001
Hysterectomy type							
LAVH	338	33.70	232	42.11	106	23.45	<0.001
TLH	665	66.30	319	57.89	346	76.55	
Vault suture material							
PDS	1	0.10	1	0.18	0	0.00	<0.001
V-lock	117	11.67	37	6.72	80	17.70	
Vicryl	797	79.46	435	78.95	362	80.09	
Other	88	8.77	78	14.16	10	2.21	

H, hysterectomy; RS, robot-assisted surgery group; LS, conventional laparoscopic surgery group; BSO, bilateral salpingo-oophorectomy; BPLD, bilateral pelvic lymph node dissection; PALD, para-aortic lymph node dissection; LAVH, laparoscopically assisted vaginal hysterectomy; TLH, total laparoscopic hysterectomy; PDS, polydioxanone suture; LN, lymph node.

Significantly less blood loss was observed during surgery in the RS group compared to the LS group (111.8 cc *vs*. 139.4 cc; P < 0.001). However, this reduction in blood loss did not result in a significant change in hemoglobin levels, likely due to minimal bleeding in both groups. There was no difference in the incidence of intraoperative complications between the two groups. Compared with the LS group, postoperative complications were significantly less in the RS group compared with the LS group (7.7% *vs*. 13.8%; *P* = 0.002), especially postoperative fever (3.1% *vs*. 8.9% in the LS and RS groups, respectively; *P* < 0.001) (**Table 4**). **Supplementary Table 4** presents the surgical results according to the number of ports utilized. Detailed Grade 3 postoperative complications are presented in **Supplementary Table 5**.

**Table 4:** Intra- and postoperative outcomes.

	Total (n = 1003)	%, SD	LS (n = 551)	%, SD	RS (n = 452)	%, SD	*P*-value
EBL, cc	127.15	152.89	139.74	159.77	111.81	142.74	<0.001
Hb change, mg/dL	-1.654	1.16	-1.7	1.15	-1.6	1.16	0.764
Length of stay, days	7.66	4.42	7.57	4.35	7.76	4.50	0.249
Intraoperative complication	24	2.4	14	2.5	10	2.2	0.735
Intraoperative transfusion	12	1.20	8	1.45	4	0.88	0.411
Bladder injury	7	0.70	2	0.36	5	1.11	0.159
Ureter injury	4	0.40	3	0.54	1	0.22	0.419
BV injury	1	0.10	0	0.00	1	0.22	0.269
Conversion to open	2	0.20	1	0.18	1	0.22	0.888
Postoperative complication	111	11.07	76	13.79	35	7.74	0.002
Fever	63	6.28	49	8.89	14	3.10	<0.001
Sepsis	0	0.00	0	0.00	0	0.00	–
VTE	0	0.00	0	0.00	0	0.00	–
Transfusion discharge	26	2.59	14	2.54	12	2.65	0.91
Transfusion 90 days	3	0.30	1	0.18	2	0.44	0.451
AKI	0	0.00	0	0.00	0	0.00	–
Pneumonia	2	0.20	2	0.36	0	0.00	0.2
Ileus	2	0.20	2	0.36	0	0.00	0.2
Wound infection	2	0.20	1	0.18	1	0.22	0.888
Vaginal complications	1	0.10	0	0.00	1	0.22	0.269
Hematoma	2	0.20	1	0.18	1	0.22	0.888
UTI	1	0.10	1	0.18	0	0.00	0.365
Bowel perforation	0	0.00	0	0.00	0	0.00	–
Abscess	2	0.20	1	0.18	1	0.22	0.888
Fistula	0	0.00	0	0.00	0	0.00	–
Wound discharge	2	0.20	0	0.00	2	0.44	0.118
VCD	5	0.50	3	0.54	2	0.44	0.82
Organ failure	0	0.00	0	0.00	0	0.00	–
Other	11	1.10	7	1.27	4	0.88	0.56

SD, standard deviation; RS, robot-assisted surgery group; LS, conventional laparoscopic surgery group; EBL, estimated blood loss; VTE, venous thromboembolism; AKI, acute kidney injury; UTI, urinary tract infection; Hb, hemoglobin; VCD, vocal cord dysfunction.

**Figure 1:**
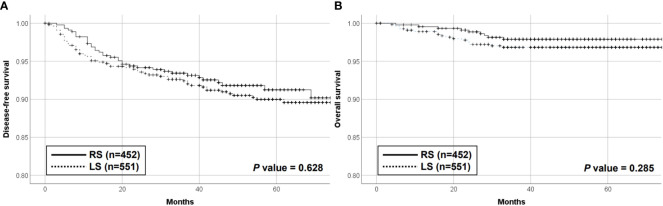
Disease-free survival **(A)** and overall survival **(B)** of the RS and LS groups. RS, robot-assisted surgery group; LS, conventional laparoscopic surgery group.

The median follow-up periods were 57 and 49 months in the RS cohort and 60 months in the LS cohort. Ninety patients (9.0%) experienced recurrence during the study period. These included 53 (9.6%) and 37 (8.2%) patients in the LS and RS groups, respectively. The median time to first recurrence was 15 and 12 months in the RS and LS groups, respectively (*P* = 0.368). Overall, 66.0% of recurrences in the LS and 70.2% in the RS cohort occurred less than 24 months after surgery (*P* = 0.608). There was no significant difference in the 5-year DFS in the RS and LS cohorts (91.2% *vs*. 90.0%, respectively; *P* = 0.628) (**Figure 1**). A total of 26 patients died during the study period, including 17 (3.1%) and 9 (2.0%) patients in the LS and RS groups, respectively. There was no significant difference in the 5-year overall rate of 97.9% *vs*. 96.8% for the RS and the LS groups, respectively (*P* = 0.285) (**Figure 1**). No significant variations were observed in the DFS and OS between the mLS, sLS, mRS, and sRS groups, as per the data presented in the **Supplementary Figure 1**. The 5-year DFS rates in the mLS, sLS, mRS, and sRS groups were 89.2%, 93.6%, 90.6%, and 100.0%, respectively. Similarly, the 5-year OS rates in the mLS, sLS, mRS, and sRS groups were 96.6%, 97.9%, 97.7%, and 100.0%, respectively (**Supplementary Figure 1**).

Cox proportional hazards regression models demonstrated that the mode of surgery was not associated with DFS (HR, 0.897; confidence interval [CI], 0.563–1.429; *P* = 0.648) or OS (HR, 0.791; CI, 0.330–1.895; *P* = 0.598) after adjusting for confounding factors including age, comorbidity, FIGO stage, grade, and LVSI (**Table 5**).

**Table 5:** Univariate and multivariate analysis of various factors for progression-free survival and overall survival.

	No. of patients	DFS				OS			
		Univariate analysis		Multivariate analysis		Univariate analysis		Multivariate analysis	
		HR (95% CI)	P	HR (95% CI)	P	HR (95% CI)	P	HR (95% CI)	P
Age, years (continuous)	1003	1.051 (1.029–1.073)	<0.001	1.035 (1.009–1.061)	0.007	1.081 (1.041–1.124)	<0.001	1.049 (1.001–1.099)	0.045
Comorbidity									
No	705	1 (Reference)	0.643	1 (Reference)	0.042	1 (Reference)	0.070	1 (Reference)	0.717
Yes	299	1.115 (0.703–1.769)		1.738 (1.019–2.962)		2.040 (0.943–4.410)		0.848 (0.347–2.068)	
FIGO stage									
I	854	1 (Reference)	<0.001	1 (Reference)	0.001	1 (Reference)	<0.001	1 (Reference)	0.004
II	47	3.692 (1.809–7.535)		2.810 (1.275–6.191)		7.029 (1.865–26.497)		4.977 (1.221–20.281)	
III	87	6.843 (4.306–10.875)		2.614 (1.446–4.726)		14.406 (5.793–35.825)		4.146 (1.371–12.541)	
IV	7	22.769 (9.020–57.473)		6.370 (2.208–18.377)		86.054 (25.804–286-979)		14.426 (3.293–63.196)	
Tumor grade									
1	609	1 (Reference)	<0.001	1 (Reference)	0.553	1 (Reference)	<0.001	1 (Reference)	0.303
2	249	2.393 (1.410–4.060)		1.197 (0.666–2.150)		2.424 (0.782–7.514)		0.747 (0.211–2.641)	
3	137	5.477 (3.285–9.133)		1.413 (0.758–2.634)		10.984 (4.221–28.586)		1.656 (0.521–5.262)	
Histology									
Endometrioid	899	1 (Reference)	<0.001	1 (Reference)	<0.001	1 (Reference)		1 (Reference)	0.023
Non-endometrioid	104	6.863 (4.487–10.496)		2.795 (1.603–4.873)		11.192 (5.174–24.213)		2.997 (1.164–7.714)	
LVSI									
No	687	1 (Reference)	<0.001	1 (Reference)		1 (Reference)	<0.001	1 (Reference)	0.041
Yes	156	5.897 (3.818–9.110)		2.356 (1.366–4.064)	0.002	10.692 (4.648–24.597)		2.908 (1.0147–8.078)	
Mode of surgery									
LS	551	1 (Reference)	0.629	1 (Reference)	0.648	1 (Reference)	0.289	1 (Reference)	0.598
RS	452	0.901 (0.592–1.373)		0.897 (0.563–1.429)		0.646 (0.288–1.449)		0.791 (0.330–1.895)	

DFS, disease-free survival; OS, overall survival; HR, hazard ratio; CI, confidence interval; FIGO, Fédération Internationale de Gynécologie et d’Obstétrique; LVSI, lymphovascular space invasion; RS, robot-assisted surgery group; LS, conventional laparoscopic surgery group.

## 4 Discussion

### 4.1 Principle findings

This study compared the long-term oncologic outcomes between RS and conventional LS in the era of a shift in the standard of care in endometrial cancer management toward MIS, after the introduction of robotic surgery in the field of gynecologic oncology (12). We observed that RS did not compromise survival outcomes when compared with conventional LS for endometrial cancer. In addition, RS was associated with significantly fewer postoperative complications when compared with LS. To the best of our knowledge, this is the largest multicenter study to compare oncologic and operative outcomes according to the modes of surgery used for the treatment of endometrial cancer in this shifting era of surgical procedures.

### 4.2 Results in the context of what is known

MIS, which includes laparoscopy and robotic surgical approaches, has significantly improved the management of endometrial cancer, and has largely replaced open surgery. MIS offers various advantages, such as reducing intra- and postoperative complications, improving patient satisfaction, and demonstrating cost efficacy (13). The introduction of robotic surgery has further enhanced surgical precision, visualization, and maneuverability, resulting in lower blood loss, reduced postoperative complications, and comparable oncological outcomes. This advancement has led to notable changes in real-world practice, particularly in high-volume hospitals. While two randomized controlled trials have reported reduced surgical complications with MIS in endometrial cancer, there is limited data available for a direct comparison between RS and LS for this condition (14, 15).

Our study encompassing 1,003 Korean patients demonstrated that patients who underwent RS were significantly less likely to develop postoperative complications as compared with those who underwent LS, which is in accordance with the results of previous studies (8, 16). This could be due to the advantages of RS, including binocular three-dimensional view, additional degrees of rotational freedom, and decreased reliance on skilled assistance. Thus, RS is gentler, causes only minor damage to the internal organs, produces less postoperative pain, and aids in faster return to a normal diet and ambulation (17, 18). Moreover, in the RS group, para-aortic lymph node dissection or sentinel lymph node sampling, which is important for staging, was performed more frequently than that in the LS group. This could be due to the Firefly technology of the robotic system (19, 20).

### 4.3 Clinical implications

Strict evaluation is necessary for any changes in the surgical approach for cancer patients to ensure that the long-term survival outcomes are not compromised. Regarding the survival outcomes of MIS for endometrial cancer, most of the available studies compared laparotomic surgery with LS rather than with RS and demonstrated that laparoscopy could be a non-inferior alternative to the traditional laparotomic approach (3, 21). Several previous studies have reported comparative survival outcomes between RS and LS for endometrial cancer (22–28). Cardenas-Goicoechea et al. observed that there were no significant differences in survival between the RS and LS cohorts (3-year PFS was 88.4% and 83.3% and 3-year OS was 93.6% and 93.3% for the LS and RS groups, respectively) (27). Similarly, Corrado et al. demonstrated that the 3-year OS was 88.4% and 91.5% and the 3-year PFS was 91.7% and 91.5% for the LS and RS groups, respectively (26). Additionally, Brudie et al. noted a 3-year PFS of 89.3% and 3-year OS of 89.1% (28), and Kilgore et al. reported a 5-year OS of 89.1% (29) in patients who underwent RS for endometrial cancer. However, the previous studies had follow-up periods ranging from 17.7 months to 47 months. These durations may have been insufficient to detect significant proportions of the DFS and OS events, unlike our study, which had a longer follow-up period of 57 months.

In Korea, the first Ministry of Food and Drug Safety approval of the da Vinci Surgical system for hysterectomy was granted in 2005, and these devices have been utilized in clinical practice since 2006 (30).

### 4.4 Research implications

A previous Korean nationwide cohort study encompassing 5,065 patients from 2012 to 2016 provided evidence that RS is a safe surgical alternative to LS, demonstrating comparable survival outcomes (5-year PFS, 93.1%; 5-year OS, 94.8%) in the RS group (5). Nevertheless, variables such as surgical stage or cell types, which may influence the oncologic outcomes of endometrial cancer, could not be considered in population-based research.

Our study included all the detailed clinical and pathologic variables from high-volume hospitals where robotic surgery procedures are actively performed, indicating that the adoption of RS did not compromise the long-term survival outcomes compared with conventional MIS after adjusting for factors including age, comorbidity, FIGO stage, grade, and LVSI.

### 4.5 Strengths and limitations

The strengths of our study are its long-term follow-up and large sample size. Although randomized controlled trials have excellent internal validity, they do not necessarily determine the impact of a specific surgical method in real-world patients as frail subgroups tend to be excluded owing to the strict inclusion criteria for enrollment (31). We believe that our data are reliable for comparing the operative and oncologic outcomes in the real clinical practice environment. This is because we analyzed well-collected clinicopathological data of unselected patients from the five largest hospitals in Korea, where both RS and LS were actively performed for endometrial cancer. Meanwhile, the retrospective nature of our study and unmeasured confounding variables are the major limitations of this study. Due to the collection of data from five different hospitals, there were substantial differences in patient characteristics, such as age, comorbidity, and performance state, making it challenging to directly accept the survival rate comparison. Consequently, it was necessary to rely on Cox regression multivariate analysis to make reasonable estimations. Potential selection bias, especially owing to the selection of patients who can afford to undergo RS, may also exist. The results of the current study should be interpreted in the context of these limitations.

Robot-assisted surgery for endometrial cancer exhibits comparable long-term survival outcomes but a lower occurrence of postoperative minor complications compared to conventional laparoscopic surgery.

## Data availability statement

The original contributions presented in the study are included in the article/**Supplementary Material**. Further inquiries can be directed to the corresponding author.

## Ethics statement

The studies involving human participants were reviewed and approved by Yonsei university of Korea (Approval No. 4-2021-0988). Written informed consent was not provided because the requirement of informed consent was waived due to the retrospective nature of the study.

## Author contributions

KE: writing original draft, review, and editing. T-JK: conceptualization, data curation, resources, and editing. J-YP: data curation, and resources. HK: data curation, resources, and editing. JP: data curation, resources, and editing. YK: conceptualization, data curation, resources, supervision, writing, and editing. All authors contributed to the article and approved the submitted version.
