# Supplementary material for: Robot-assisted versus conventional laparoscopic surgery for endometrial cancer: long-term comparison of outcomes
Source: Front Oncol. 2023 Sep 15;13:1219371. doi: 10.3389/fonc.2023.1219371 (PMC10540847; doi:10.3389/fonc.2023.1219371)
Supplement: Supplementary file 1 [file DataSheet_1.docx]

Supplement Table 1. Patient characteristics in relation to the number of ports utilized.

|  | Total (n=1003) | %, SD | Multiport Laparoscopy (n=456) | %, SD | Single port Laparoscopy (n=95) | %, SD | Multiport Robot-assisted (n=419) | %, SD | Single port Robot-assisted* (n=33) | %, SD | P-value |
| --- | --- | --- | --- | --- | --- | --- | --- | --- | --- | --- | --- |
| Age, median (range) | 55 (23-86) |  | 54 (23-86) |  | 53 (34-85) |  | 52 (27-72) |  | 51 (31-69) |  | <0.001 |
| BMI, mean (SD) | 24.349 | 3.94 | 24.641 | 3.99 | 24.44 | 3.84 | 25.031 | 4.57 | 25.827 | 5.49 | 0.224 |
| Comorbidity | 299 | 29.81 | 152 | 33.33 | 31 | 32.63 | 110 | 26.25 | 6 | 18.18 | 0.052 |
| Angina | 2 | 0.20 | 2 | 0.44 | 0 | 0.00 | 0 | 0.00 | 0 | 0.00 |  |
| MI | 2 | 0.20 | 2 | 0.44 | 0 | 0.00 | 0 | 0.00 | 0 | 0.00 |  |
| Afib | 1 | 0.10 | 1 | 0.22 | 0 | 0.00 | 0 | 0.00 | 0 | 0.00 |  |
| Stroke | 5 | 0.50 | 4 | 0.88 | 0 | 0.00 | 1 | 0.24 | 0 | 0.00 |  |
| CEVD | 10 | 1.00 | 4 | 0.88 | 1 | 1.05 | 5 | 1.19 | 0 | 0.00 |  |
| PVD | 2 | 0.20 | 0 | 0.00 | 1 | 1.05 | 1 | 0.24 | 0 | 0.00 |  |
| COPD | 3 | 0.30 | 0 | 0.00 | 1 | 1.05 | 2 | 0.48 | 0 | 0.00 |  |
| CPD | 10 | 1.00 | 3 | 0.66 | 2 | 2.11 | 5 | 1.19 | 0 | 0.00 |  |
| LC | 8 | 0.80 | 2 | 0.44 | 3 | 3.16 | 3 | 0.72 | 0 | 0.00 |  |
| DLP | 63 | 6.28 | 35 | 7.68 | 2 | 2.11 | 23 | 5.49 | 3 | 9.09 |  |
| HTN | 220 | 21.93 | 112 | 24.56 | 21 | 22.11 | 83 | 19.81 | 4 | 12.12 |  |
| DIA | 63 | 6.28 | 38 | 8.33 | 6 | 6.32 | 18 | 4.30 | 1 | 3.03 |  |
| Renal | 4 | 0.40 | 4 | 0.88 | 0 | 0.00 | 0 | 0.00 | 0 | 0.00 |  |
| DEM | 2 | 0.20 | 1 | 0.22 | 0 | 0.00 | 1 | 0.24 | 0 | 0.00 |  |
| Performance state |  |  |  |  |  |  |  |  |  |  |  |
| ASA I | 417 | 41.58 | 169 | 37.06 | 35 | 36.84 | 201 | 47.97 | 12 | 36.36 | 0.001 |
| ASA II | 507 | 50.55 | 246 | 53.95 | 44 | 46.32 | 200 | 47.73 | 17 | 51.52 |  |
| ASA III | 75 | 7.48 | 38 | 8.33 | 15 | 15.79 | 18 | 4.30 | 4 | 12.12 |  |
| ASA IV | 4 | 0.40 | 3 | 0.66 | 1 | 1.05 | 0 | 0.00 | 0 | 0.00 |  |

SD, standard deviation; RS, robot-assisted surgery group; LS, conventional laparoscopic surgery group; BMI, body mass index; MI, myocardial infarction; Afib, atrial fibrillation; CVD, cerebrovascular disease; PVD, peripheral vascular disease; COPD, chronic obstructive pulmonary disease; CPD, chronic pulmonary disease; LC, liver cirrhosis; HTN, hypertension; DM, diabetes with chronic complications; ASA, American Society of Anesthesiologists; *Single port Robot-assisted group includes both da Vinci Xi® Single-site® Technology and da Vinci SP® system

Supplement Table 2. Pathologic outcomes in relation to the number of ports utilized.

|  | Total (n=1003) | %, SD | Multiport Laparoscopy (n=456) | %, SD | Single port Laparoscopy (n=95) | %, SD | Multiport Robot-assisted (n=419) | %, SD | Single port Robot-assisted* (n=33) | %, SD | P-value |
| --- | --- | --- | --- | --- | --- | --- | --- | --- | --- | --- | --- |
| Stage |  |  |  |  |  |  |  |  |  |  |  |
| IA | 754 | 75.17 | 338 | 74.12 | 68 | 71.58 | 320 | 76.37 | 28 | 84.85 | 0.85 |
| IB | 105 | 10.47 | 54 | 11.84 | 8 | 8.42 | 39 | 9.31 | 4 | 12.12 |  |
| II | 47 | 4.69 | 22 | 4.82 | 6 | 6.32 | 19 | 4.53 | 0 | 0.00 |  |
| IIIA | 24 | 2.39 | 12 | 2.63 | 3 | 3.16 | 9 | 2.15 | 0 | 0.00 |  |
| IIIB | 4 | 0.40 | 3 | 0.66 | 0 | 0.00 | 1 | 0.24 | 0 | 0.00 |  |
| IIIC1 | 33 | 3.29 | 13 | 2.85 | 4 | 4.21 | 15 | 3.58 | 1 | 3.03 |  |
| IIIC2 | 29 | 2.89 | 13 | 2.85 | 4 | 4.21 | 12 | 2.86 | 0 | 0.00 |  |
| IVB | 7 | 0.70 | 1 | 0.22 | 2 | 2.11 | 4 | 0.95 | 0 | 0.00 |  |
| Histology |  |  |  |  |  |  |  |  |  |  |  |
| Endometrioid | 899 | 89.63 | 398 | 87.28 | 88 | 92.63 | 381 | 90.93 | 32 | 96.97 | 0.098 |
| Non-endometrioid | 104 | 10.37 | 58 | 12.72 | 7 | 7.37 | 38 | 9.07 | 1 | 3.03 |  |
| Grade |  |  |  |  |  |  |  |  |  |  |  |
| G1 | 609 | 60.72 | 274 | 60.09 | 59 | 62.11 | 253 | 60.38 | 23 | 69.70 | 0.62 |
| G2 | 249 | 24.83 | 117 | 25.66 | 22 | 23.16 | 101 | 24.11 | 9 | 27.27 |  |
| G3 | 137 | 13.66 | 59 | 12.94 | 14 | 14.74 | 63 | 15.04 | 1 | 3.03 |  |
| NA | 8 | 0.80 | 6 | 1.32 | 0 | 0.00 | 2 | 0.48 | 0 | 0.00 |  |
| LVSI | 156 | 15.55 | 71 | 12.89 | 20 | 21.05 | 61 | 14.56 | 4 | 12.12 | 0.677 |
| Invasion depth, cm (SD) | 0.451 | 0.67 | 0.478 | 0.65 | 0.485 | 0.63 | 0.422 | 0.71 | 0.346 | 0.47 | 0.46 |
| Tumor size, cm (SD) | 2.055 | 1.88 | 2.058 | 1.87 | 2.268 | 1.81 | 2.061 | 1.92 | 1.309 | 1.69 | 0.093 |

SD, standard deviation; RS, robot-assisted surgery group; LS, conventional laparoscopic surgery group; FIGO, Fédération Internationale de Gynécologie et d'Obstétrique; NA, not available; LVSI, lymphovascular space invasion; *Single port Robot-assisted group includes both da Vinci Xi® Single-site® Technology and da Vinci SP® system

Supplement Table 3. Operative description in relation to the number of ports utilized.

|  | Total (n=1003) | % | Multiport Laparoscopy (n=456) | % | Single port Laparoscopy (n=95) | % | Multiport Robot-assisted (n=419) | % | Single port Robot-assisted* (n=33) | % | P-value |
| --- | --- | --- | --- | --- | --- | --- | --- | --- | --- | --- | --- |
| Op type |  |  |  |  |  |  |  |  |  |  |  |
| Hysterectomy only | 5 | 0.50 | 3 | 0.66 | 0 | 0.00 | 2 | 0.48 | 0 | 0.00 | <0.001 |
| H+BS | 26 | 2.59 | 12 | 2.63 | 9 | 9.47 | 5 | 1.19 | 0 | 0.00 |  |
| H+BSO | 43 | 4.29 | 27 | 5.92 | 10 | 10.53 | 6 | 1.43 | 0 | 0.00 |  |
| H+BSO+BPLD | 403 | 40.18 | 198 | 43.42 | 10 | 10.53 | 171 | 40.81 | 24 | 72.73 |  |
| H+BSO+BPLD+PALD | 490 | 48.85 | 195 | 42.76 | 60 | 63.16 | 226 | 53.94 | 9 | 27.27 |  |
| H+BSO+BPLD+PALD+Omentectomy | 36 | 3.59 | 21 | 4.61 | 6 | 6.32 | 9 | 2.15 | 0 | 0.00 |  |
| Hysterectomy type |  |  |  |  |  |  |  |  |  |  |  |
| LAVH | 338 | 33.70 | 220 | 48.25 | 12 | 12.63 | 92 | 21.96 | 14 | 42.42 | <0.001 |
| TLH | 665 | 66.30 | 236 | 51.75 | 83 | 87.37 | 327 | 78.04 | 19 | 57.58 |  |
| Vault suture material |  |  |  |  |  |  |  |  |  |  |  |
| PDS | 1 | 0.10 | 1 | 0.22 | 0 | 0.00 | 0 | 0.00 | 0 | 0.00 | <0.001 |
| V-lock | 117 | 11.67 | 31 | 6.80 | 6 | 6.32 | 74 | 17.66 | 6 | 18.18 |  |
| Vicryl | 797 | 79.46 | 391 | 85.75 | 44 | 46.32 | 339 | 80.91 | 23 | 69.70 |  |
| Other | 88 | 8.77 | 33 | 7.24 | 45 | 47.37 | 6 | 1.43 | 4 | 12.12 |  |
| Sentinel LN biopsy | 365 | 36.39 | 55 | 12.06 | 48 | 50.53 | 231 | 55.13 | 31 | 93.94 | <0.001 |

H, hysterectomy; RS, robot-assisted surgery group; LS, conventional laparoscopic surgery group; BSO, bilateral salpingo-oophorectomy; BPLD, bilateral pelvic lymph node dissection; PALD, para-aortic lymph node dissection; LAVH, laparoscopically assisted vaginal hysterectomy; TLH, total laparoscopic hysterectomy; PDS, polydioxanone suture; LN, lymph node; *Single port Robot-assisted group includes both da Vinci Xi® Single-site® Technology and da Vinci SP® system

Supplement Table 4. Surgical results in relation to the number of ports utilized.

|  | Total (n=1003) | %, SD | Multiport Laparoscopy (n=456) | %, SD | Single port Laparoscopy (n=95) | %, SD | Multiport Robot-assisted (n=419) | %, SD | Single port Robot-assisted* (n=33) | %, SD | P-value |
| --- | --- | --- | --- | --- | --- | --- | --- | --- | --- | --- | --- |
| EBL | 127.15 | 152.89 | 155.16 | 168.61 | 65.68 | 71.08 | 109.29 | 137.45 | 143.79 | 198.07 | <0.001 |
| Hb change | -1.654 | 1.16 | -1.69 | 1.18 | -1.74 | 1.00 | -1.62 | 1.14 | -1.36 | 1.44 | 0.31 |
| Length of stay | 7.66 | 4.42 | 7.52 | 4.11 | 7.83 | 5.37 | 7.87 | 4.61 | 6.42 | 2.51 | 0.249 |
| Intraoperaive complication | 24 | 2.40 | 11 | 2.41 | 3 | 3.16 | 9 | 2.15 | 1 | 3.03 | 0.939 |
| IOTRANSF | 12 | 1.20 | 6 | 1.32 | 2 | 2.11 | 4 | 0.95 | 0 | 0.00 | 0.723 |
| BLADDER_injury | 7 | 0.70 | 1 | 0.22 | 1 | 1.05 | 4 | 0.95 | 1 | 3.03 | 0.198 |
| URETER_injury | 4 | 0.40 | 3 | 0.66 | 0 | 0.00 | 1 | 0.24 | 0 | 0.00 | 0.67 |
| BV_injury | 1 | 0.10 | 0 | 0.00 | 0 | 0.00 | 1 | 0.24 | 0 | 0.00 | 0.707 |
| Conversion | 2 | 0.20 | 1 | 0.22 | 0 | 0.00 | 1 | 0.24 | 0 | 0.00 | 0.961 |
| Postoperative complication | 111 | 11.07 | 66 | 14.47 | 10 | 10.53 | 30 | 7.16 | 5 | 15.15 | 0.006 |
| FEVER | 49 | 4.89 | 44 | 9.65 | 5 | 5.26 | 11 | 2.63 | 3 | 9.09 | <0.001 |
| SEPSIS | 0 | 0.00 | 0 | 0.00 | 0 | 0.00 | 0 | 0.00 | 0 | 0.00 |  |
| VTE | 0 | 0.00 | 0 | 0.00 | 0 | 0.00 | 0 | 0.00 | 0 | 0.00 |  |
| TRANSF_DISCH | 14 | 1.40 | 11 | 2.41 | 3 | 3.16 | 11 | 2.63 | 1 | 3.03 | 0.977 |
| TRANSF_90D | 1 | 0.10 | 1 | 0.22 | 0 | 0.00 | 2 | 0.48 | 0 | 0.00 | 0.819 |
| AKI | 0 | 0.00 | 0 | 0.00 | 0 | 0.00 | 0 | 0.00 | 0 | 0.00 |  |
| PNEUM | 2 | 0.20 | 2 | 0.44 | 0 | 0.00 | 0 | 0.00 | 0 | 0.00 | 0.493 |
| ILEUS | 2 | 0.20 | 2 | 0.44 | 0 | 0.00 | 0 | 0.00 | 0 | 0.00 | 0.493 |
| WOUNDINF | 1 | 0.10 | 1 | 0.22 | 0 | 0.00 | 1 | 0.24 | 0 | 0.00 | 0.961 |
| VAG | 0 | 0.00 | 0 | 0.00 | 0 | 0.00 | 1 | 0.24 | 0 | 0.00 | 0.707 |
| HEM | 1 | 0.10 | 1 | 0.22 | 0 | 0.00 | 1 | 0.24 | 0 | 0.00 | 0.961 |
| UTI | 1 | 0.10 | 1 | 0.22 | 0 | 0.00 | 0 | 0.00 | 0 | 0.00 | 0.753 |
| BP | 0 | 0.00 | 0 | 0.00 | 0 | 0.00 | 0 | 0.00 | 0 | 0.00 |  |
| ABSCESS | 1 | 0.10 | 0 | 0.00 | 1 | 1.05 | 1 | 0.24 | 0 | 0.00 | 0.214 |
| FISTULA | 0 | 0.00 | 0 | 0.00 | 0 | 0.00 | 0 | 0.00 | 0 | 0.00 |  |
| WOUNDDISR | 0 | 0.00 | 0 | 0.00 | 0 | 0.00 | 2 | 0.48 | 2 | 6.06 | 0.425 |
| VCD | 3 | 0.30 | 3 | 0.66 | 0 | 0.00 | 2 | 0.48 | 0 | 0.00 | 0.831 |
| ORGFAIL | 0 | 0.00 | 0 | 0.00 | 0 | 0.00 | 0 | 0.00 | 0 | 0.00 |  |
| AEOTHER | 7 | 0.70 | 6 | 1.32 | 1 | 1.05 | 3 | 0.72 | 1 | 3.03 | 0.593 |

SD, standard deviation; RS, robot-assisted surgery group; LS, conventional laparoscopic surgery group; EBL, estimated blood loss; VTE, venous thromboembolism; AKI, acute kidney injury; UTI, urinary tract infection; Hb, hemoglobin; VCD, vocal cord dysfunction: *Single port Robot-assisted group includes both da Vinci Xi® Single-site® Technology and da Vinci SP® system

Supplement Table 5. Detailed Grade 3 postoperative complication

|  | Total (n=1003) | % | Laparoscopy (n=551) | % | Robot-assisted (n=452) | % | P-value |
| --- | --- | --- | --- | --- | --- | --- | --- |
| Grade 3 complications | 16 | 1.60 | 9 | 1.60 | 7 | 1.50 | 0.915 |
| Detailed complications |  |  | *Vault dehiscence (n=1) *Vaginal vault bleeding (n=2)  *Ureter injury (n=2) *Postoperative intraabdominal bleeding (n=2) *Pneumothorax (n=1) *Lymphocele (n=1) | | *Vault dehiscence (n=2) *Operative site hernia (n=1) *Operative site rupture (n=2) *Bladder rupture (n=1) *Rectal perforation (n=1) | |  |

Supplement Figure 1. Disease-free survival and overall survival in mLS, sLS, mRS, and sRS groups.


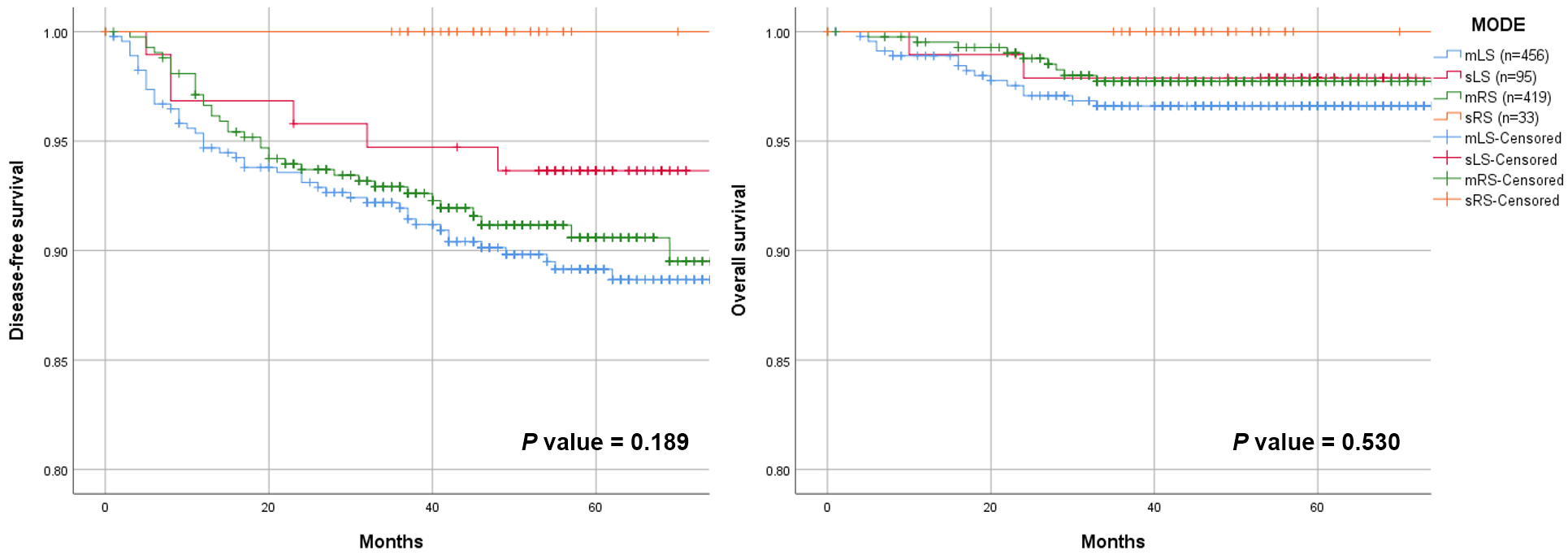


mLS. Multiport Laparoscopy; sLS, Single port Laparoscopy; mRS, Multiport Robot-assisted; sRS, Single port Robot-assisted*; Single port Robot-assisted group includes both da Vinci Xi® Single-site® Technology and da Vinci SP® system
